# Reactant pairs and reaction organization patterns produced by a new rule-based approach

**DOI:** 10.1186/s13104-018-3724-8

**Published:** 2018-08-24

**Authors:** Carlos Vazquez-Hernandez, Antonio Loza, Rosa-Maria Gutierrez-Rios

**Affiliations:** Departamento de Microbiología Molecular, Instituto de Biotecnología Universidad Nacional Autónoma de México, Apdo. Postal 510-3, 62250 Cuernavaca, Morelos Mexico

**Keywords:** Metabolic reaction, Reaction patterns, Reactant pairs, Compound pairs

## Abstract

**Objectives:**

Improvements in bioinformatics applications for the enzyme identification of biochemical reactions, enzyme classifications, mining for specific inhibitors and pathfinding require the accurate computational detection of reaction similarity. We provide a set of substrate-product pairs, clustered by reactions that share similar chemical transformation patterns, for which accuracy was calculated, comparing this set with manually curated data sets.

**Data description:**

The data were analyzed by a new method that naturally split each reaction into compound pairs and loner compounds, which we called architectures (Vazquez-Hernandez et al. in BMC Syst Biol 12:63, 2018). The data include a set of 7491 curated reactions from the KEGG-Ligand data set. The data are presented in two formats, a string format and a tree structure, both of which reflect the splitting process and the final architectures of each reaction. We are also reporting sets of reactions that show similar splitting patterns naturally grouped into clusters of tree structures. The compound pairs in each cluster were compared with the reactant pairs proposed by the KEGG-RCLASS data set, and a match precision value is also provided. These data were collected with the aim of providing research with a confident set of reactant pairs that is useful for selecting between alternative substrate-product pairs predicted by pathfinders.

## Objective

Genome-scale metabolic reconstruction requires that information about chemical transformations be known, and atom mappers are convenient methods for providing a one-to-one comparison of an atom in a substrate and an atom in a product [1, 2]. Atom mappers use heuristic approximations to rapidly identify common substructures between two compounds on the basis of a graph comparison method [2–4], information on the chemical environment and the removal of noninformative atoms. As a result, atom mappers can give optimal and suboptimal solutions that must be manually confirmed to ensure their accuracy. Most importantly, previous work related to atom mappers has focused on how to efficiently compute metrics for chemical structures, but the accuracy of these methods has not been assessed for large networks [1, 3]. This last point is an important issue because methods devoted to pathway discovery have used the results of atom mapping and reactant pairings as input to define new pathways. Faust et al. [5] demonstrated this point by computing the best curated KEGG pairs with a weighting scheme penalizing highly connected compounds, which improved the performance of pathfinding methods.

These observations inspired us to construct a method able to identify architectures (“pairs” and “loner” compounds) that uses a minimum of chemical information and does not remove any of the compounds or atoms in a reaction, such that its results avoid “manual curation” as much as possible. For this purpose, we performed a statistical comparison of the tree structure pairs (TS pairs) proposed by our method and those in the RPAIR/RCLASS data sets, which gave as a result a precision number that can be interpreted as the confidence between the predicted set of reactant pairs from RPAIR/RCLASS and TS pairs [6]. In this note, we present the TS pairs, the clusters of TS pairs (CTSs) and the precision value for each reaction grouped in each CTS.

## Data description

### Tree structure pairs

We are reporting TS pairs (substrate-product pairs) proposed by our method for 7491 curated reactions that are completely described in the Kyoto Encyclopedia of Genes and Genomes (KEGG)-Ligand data set [7]. The data included reactions that are completely described in data sets stored in the 2015 version of the KEGG knowledgebase. From the COMPOUND data set, we collected the IDs, chemical formulas and molecular weights of 7661 compounds. We limited our analysis to a well-curated and verifiable set, and all reactions that included compounds from the GLYCAN data set and reactions with coefficients and subscripts that had not been completely described were removed. The method used to generate the TS pairs and loner compounds is fully described in Ref. [6]. A copy of the code is also provided as part of the results presented in this manuscript (Table 1).

**Table 1:** Overview of the data files

Label	Name of data file/data set	File types (file extension)	Data repository identifier (DOI)
Data file1	Compound pairs with a precision value [8]	Text file (.txt)	Figshare 10.6084/m9.figshare.6768449
Data file 2	Compound pairs without a precision value [9]	Text file (.txt)	Figshare 10.6084/m9.figshare.6789899
Data file 3	Reaction splitting using the balance rule [10]	Text file (.txt)	Figshare 10.6084/m9.figshare.6789902
Data file 4	Reaction splitting using the count rule [11]	Text file (.txt)	Figshare 10.6084/m9.figshare.6789905
Data file 5	Reaction splitting using the both rules [12]	Text file (.txt)	Figshare 10.6084/m9.figshare.6789911
Data file 6	RPAIR/RCLASS [7, 13]	Text file (.txt)	Figshare 10.6084/m9.figshare.6967439
Data file 7	reaCTS software [14]	Perl library (.pm)	Figshare 10.6084/m9.figshare.6789914
Data file 8	CurateKEGG [15]	Perl library (.pm)	Figshare 10.6084/m9.figshare.6789917

### Architectures and tree structure patterns

The organization patterns of pair and loner compounds for each reaction are provided. For every reaction in the data set, we constructed a TS. We used Perl scripts to construct an algorithm based on the calculated mass differences and the frequencies of Cartesian products in the metabolic network to divide each reaction in the data set into compound pairs and loner compounds. For this purpose, we created two rules, the balance and count rules. The implementation and use of these rules are described in detail in the methods section of the original paper [6]. The algorithm is capable of giving the pairs and/or loner compounds associated with each reaction in an organized fashion, automatically creating a reaction pattern. The algorithm also provides the rule applied to generate each architecture as the order and origin (set of compounds within the reaction) from which each architecture was obtained. We obtained a tree structure that shows the reaction pattern and its partition history.

After the successive application of the rules, we constructed a representation visualized as a tree [6]. We also represented each TS in a JSON (JavaScript Object Notation) format and in two simplified formats (Data files 3–5). These formats are exemplified below; Eq. 1a gives a generic syntax outline, and Eqs. 1b–c specify reaction R00760, in which d-fructose is transformed in d-frutose-6-phosphate.

1a $${\text{root}}({\text{balance}}\left( {{\text{compound}}\_{\text{compound}}} \right)\left( {{\text{compound}}\_{\text{compound}}} \right)$$

1b $${\text{root}}\left( {{\text{balance}}\left( {{\text{C}}000 9 5\_{\text{C}}000 8 5} \right)\left( {{\text{C}}0000 2\_{\text{C}}0000 8} \right)} \right)$$

1c $$> \left( {!\left( {{\text{C}}\_{\text{C}}} \right)\left( {{\text{C}}\_{\text{C}}} \right)} \right)$$

### Clusters of tree structures

For each reaction, a TS was proposed, and the architectures found were represented as in Eq. 1c. The TSs available for each reaction were clustered into CTSs according to their topology. We are providing the 71 groups that show the reaction patterns clustered by their similarity on chemical transformations. Using a Bayesian test (described in detail in the original manuscript) on the first 22 CTSs, we included their precision level when compared with each RPAIR in the RPAIR/RCLASS data set [6, 7].

## Limitations

In the manuscript, we did not show the entire list of TS pairs or CTSs yielded by the method [3].

- A statistical precision value could not be generated for 49 CTSs because they had fewer than 10 elements (CTSs from 23 to 71).
- The reactions and TS pairs that do not have a concordant pair in the RCLASS need manual curation.
- In contrast to the RPAIR data set, our method does not allow us to pair a compound more than one time with another for the same reaction.

## Abbreviations

TS: tree structure
CTS: cluster of tree structures
